# Clinically Significant Carbapenemases in Gram-Negative Pathogens: Molecular Diversity and Advances in β-Lactamase Inhibitor Therapy

**DOI:** 10.3390/antibiotics15040413

**Published:** 2026-04-18

**Authors:** Jessi M. Grossman, Dorothea K. Thompson

**Affiliations:** 1 South College School of Pharmacy, Knoxville, TN 37922, USA; jessimgross@gmail.com; 2 Department of Pharmaceutical Sciences, South College School of Pharmacy, Knoxville, TN 37922, USA

**Keywords:** carbapenem-resistant Gram-negative bacteria, carbapenemases, *bla* gene diversity, serine carbapenemases (SME, IMI/NMC-A, KPC, OXA), metallo-β-lactamases (IMP, VIM, NDM), β-lactam/β-lactamase inhibitors, novel investigational β-lactamase inhibitors, resistance

## Abstract

Carbapenems comprise a class of β-lactam antibiotics with broad-spectrum hydrolytic activity and are often reserved as last-line agents for the treatment of serious multidrug-resistant (MDR) bacterial infections. Clinically important nosocomial MDR Gram-negative bacteria (GNB) include *Klebsiella pneumoniae*, *Pseudomonas aeruginosa*, and *Acinetobacter baumannii*. Carbapenem resistance among these organisms is predominantly mediated by the production of β-lactamases called carbapenemases, such as *K. pneumoniae* carbapenemase (KPC), New Delhi metallo-β-lactamase (NDM), imipenemase (IMP), Verona integron-encoded metallo-β-lactamase (VIM), and selected oxacillinase (OXA)-type carbapenemases. These enzymes degrade carbapenems, significantly compromising their clinical efficacy. To address escalating antimicrobial resistance, novel next-generation β-lactamase inhibitors (BLIs), partnered with established β-lactams (BLs), have been approved or are currently under development to inhibit carbapenemase activity. The present narrative review aims to synthesize the most current information on the major carbapenemases and discusses recently approved and investigational BL/BLI combination therapies in terms of their mechanisms of action, spectrum of activity, gaps in coverage, and available clinical and in vitro evidence. Development of resistance to novel BL/BLI combinations is also examined. Comparative analysis of inhibitory spectra and microbiological coverage indicates a continued need for metallo-β-lactamase inhibitors with direct pan-inhibitory activity, pathogen-specific BL/BLI regimens for carbapenem-resistant *A. baumannii*, and carbapenemase-targeted agents effective in the context of non-enzymatic resistance mechanisms. Treatment-emergent resistance to novel BL/BLIs and limitations in activity profiles underscore the critical need for continued innovation in pipeline development, vigilant global and local surveillance of carbapenemase epidemiology, and robust antimicrobial stewardship strategies to aid in preserving the efficacy of the antibacterial drug armamentarium.

## 1. Introduction

In 2024, the World Health Organization (WHO) revised its previous Bacterial Priority Pathogens List (BPPL), categorizing 24 antibiotic-resistant bacterial pathogens into three priority tiers (critical, high, and medium) for the purpose of guiding prioritization of research, investments, and drug development. Bacterial pathogens were assessed based on such criteria as mortality, incidence, 10-year resistance trends, treatability, and antibacterial pipeline status [1]. Most notably, carbapenem-resistant *Acinetobacter baumannii* (CRAB) and carbapenem-resistant Enterobacterales (CRE) were ranked as being of critical priority, while carbapenem-resistant *Pseudomonas aeruginosa* (CRPA) was assigned high-priority status [1]. The top-ranked bacterial pathogen in terms of urgent health threats was carbapenem-resistant *Klebsiella pneumoniae* (CRKP) [1]. The WHO BPPL study highlights the substantial public health burden of carbapenem-resistant Gram-negative bacteria (CR-GNB) and the critical need for novel interventions and therapeutic modalities.

**Figure 1:**
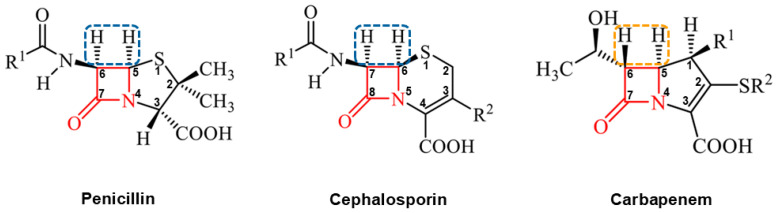
Core chemical structures of penicillin, cephalosporin, and carbapenem β-lactam antibiotic classes. The common β-lactam ring is depicted in red. Stereochemical differences include the *cis* configuration (indicated by blue dashes) of penicillin and cephalosporin between the β-lactam ring and the adjacent ring, in contrast to the *trans* configuration (denoted by gold dashes) at this position in carbapenems.

The emergence and rapid dissemination of carbapenem resistance among clinically relevant GNB is a serious threat to human health worldwide. Collectively, carbapenems (e.g., meropenem, doripenem, ertapenem, and imipenem) comprise a subclass of β-lactam antibiotics that are distinguished by the broadest spectrum of antibacterial activity among agents that inhibit bacterial cell wall biosynthesis by covalently inactivating D-transpeptidases (penicillin-binding proteins, PBPs) [2]. As shown in Figure 1, the *trans* configuration between the substituent at C-5 of the bicyclic core and the hydroxyethyl R_2_ side chain at C-6 allows carbapenems to resist hydrolysis by many β-lactamases, in contrast to the *cis* configuration of penicillins and cephalosporins [3]. Because of their stability and bactericidal potency, carbapenems have been reserved historically as the last therapeutic drug of choice to treat complicated multidrug-resistant (MDR) infections caused by bacterial pathogens with resistance phenotypes to penicillin and cephalosporins [4,5,6,7]. However, the growing prevalence of CR-GNB, particularly in healthcare settings, significantly limits the therapeutic options available for effectively managing MDR infections [8,9].

**Figure 2:**
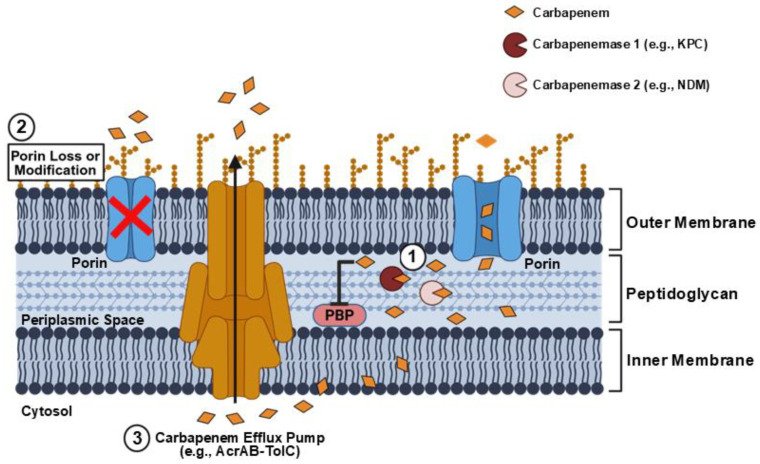
Enzymatic and Non-Enzymatic Mechanisms of Carbapenem Resistance in GNB. Gram-negative bacteria primarily utilize three mechanisms of carbapenem resistance: (1) carbapenem-hydrolyzing β-lactamases called carbapenemases, (2) decreased drug permeability due to porin loss or modification, and (3) active carbapenem extrusion from the cell because of efflux pump over-expression. (Figure created in https://BioRender.com).

The dominant mechanism driving the global rise of carbapenem resistance among GNB is the acquisition and horizontal gene transfer of carbapenemases [10,11,12,13]. Carbapenemases are a diverse family of β-lactamase enzymes that can hydrolyze and inactivate various β-lactam antibiotics, including penicillins, cephalosporins, monobactams, and carbapenems [14,15]. Non-enzymatic mechanisms of carbapenem resistance in GNB primarily involve reduced outer membrane permeability via loss of porin gene expression and/or structural alterations due to mutations in chromosomally encoded porin genes (e.g., non-selective OmpK35/OmpK36 porins in *K. pneumoniae* and OprD in *P. aeruginosa*) [16,17,18,19] and increased active drug extrusion via overexpression of genes encoding efflux pumps (e.g., AcrAB-TolC in Enterobacterales and MexAB-OprM or MexXY-OprM in *P. aeruginosa*) [20,21]. The principal enzymatic and non-enzymatic mechanisms of carbapenem resistance in GNB are illustrated in Figure 2. It is important to note that, in non-carbapenemase-producing GNB, clinically meaningful carbapenem resistance is achieved through a combined contribution of non-enzymatic mechanisms and other resistance determinants, particularly β-lactamases with limited carbapenem-hydrolyzing activity like extended-spectrum β-lactamases (ESBLs) and AmpC enzymes [22,23,24]. By contrast, carbapenemases alone are sufficient to confer a fully carbapenem-resistant phenotype in GNB that harbor these enzymes [14].

Globally, CR-GNB are a major cause of hospital-acquired infections, particularly ventilator-associated pneumonia and bacteremia in the intensive care unit (ICU), and are associated with substantial morbidity and mortality [25]. The disproportionately high presence of CR-GNB in ICU settings possibly reflects strong selective pressure due to broad-spectrum antibiotic use and enhanced transmission in these environments. A meta-analysis reported an increased relative risk (RR) of overall death (RR, 2.14, 95% Confidence Interval [CI] 1.85–2.48) among patients with CRE infections compared with those infected by carbapenem-susceptible Enterobacterales [26]. An estimated 26.3% of global bloodstream infection-related deaths in 2019 were attributable to carbapenem-resistant pathogens, predominantly driven by *A. baumannii*, *K. pneumoniae*, and *P. aeruginosa* as the principal CR-GNB contributors to mortality [27]. In a recent meta-regression study, the 30-day mortality rate was significantly higher for CRKP bacteremia compared to non-CRKP bacteremia (Odds Ratio [OR], 3.87, 95% CI 3.01–3.49) [28]. Recent studies have identified length of hospital stay, previous antibiotic exposure (especially carbapenems), mechanical ventilation, and invasive interventions like central venous catheter use as being important independent risk factors for CR-GNB infections [29,30,31]. In a multicenter retrospective cohort study, 30-day mortality rates were not significantly different for patients with carbapenemase-producing (CP) versus non-CP CRE bacteremia, although CP-CRE cases were more frequently identified in ICU settings [32]. Drawing on data from the Antimicrobial Resistance Laboratory Network of the Centers for Disease Control and Prevention (CDC), investigators estimated the annual unadjusted CRE incidence in the United States (USA) to be 18% higher in 2023 compared to 2019, while the age-adjusted incidence of CP-CRE increased 69% over the same five-year observational period [33].

This narrative review aims to synthesize the most up-to-date literature on the major clinically relevant carbapenemases, with particular emphasis on the predominant genetic variants and the species-specific diversity of carbapenemase-encoding genes in high-consequence nosocomial pathogens like *K. pneumoniae*, *P. aeruginosa*, and *A. baumannii*. The epidemiology and geographic prevalence of carbapenemases are briefly described here but not examined in depth; readers are referred to more comprehensive reviews published elsewhere [34,35,36,37]. Additionally, we discuss newly approved β-lactam/β-lactamase inhibitor (BL/BLI) combination therapies and similar agents currently in Phase 1 or 3 clinical development that are being advanced to address the increasing clinical burden of carbapenemase-producing GNB. Critical gaps in inhibitory spectra and microbiological coverage of approved and investigational next-generation BLIs are identified. The emergence of resistance to novel BL/BLI combinations is also reviewed.

## 2. Materials and Methods

A structured narrative review of the literature was performed initially in October 2024 to synthesize current knowledge on the major clinically significant carbapenemases and next-generation BL/BLI combination therapeutics. Searches were conducted in the following databases: PubMed, Scopus, Google Scholar, the National Center for Biotechnology Information (NCBI), and ClinicalTrials.gov. Updated searches were conducted periodically until 2 April 2026. Additional relevant publications were identified through manual review of reference lists from key articles and reviews. The literature review was implemented using such search terms as “carbapenemase”, “carbapenem resistance”, “genetic diversity of carbapenemases”, “KPC”, “NDM”, “IMP”, “VIM”, “carbapenem-hydrolyzing OXA”, “novel therapeutics against carbapenemase”, “novel antibiotics to treat multidrug resistance”, and “novel beta-lactamase inhibitors.” Our search strategy also included gray literature, e.g., reports from the WHO, CDC, and US Food and Drug Administration (FDA). Only articles that were relevant to the scope of this narrative review, published in English, peer-reviewed, and available in full text were included for further analysis. Preprints were excluded. Because our objective was to provide a thematic overview and targeted narrative synthesis of the literature, a formal systematic review methodology with a predefined screening process and quality assessment was not employed.

## 3. Classification and Genetic Diversity of Carbapenemases

Carbapenemases comprise a diverse family of β-lactamases that are categorized according to two distinct classification systems: Ambler system, a molecular scheme based on amino acid sequence homology [14], and the Bush-Jacoby-Medeiros system, a functional scheme based on substrate and inhibitor profiles [38,39]. Previously characterized carbapenemase enzymes belong to Ambler class A (e.g., *K. pneumoniae* carbapenemase [KPC type]), class B (e.g., Verona integron-encoded metallo-β-lactamase [VIM type], Imipenemase metallo-β-lactamase [IMP type], and New Delhi metallo-β-lactamase [NDM type]), and class D (Oxacillinase [OXA type]) [39,40]. Enzymes in Ambler classes A, C, and D share a conserved serine residue in the catalytic site that is critical for β-lactam hydrolysis, whereas class B enzymes are metallo-β-lactamases (MBLs) that use one or two active-site zinc ions to facilitate β-lactam hydrolysis [39]. The most prevalent carbapenemases identified globally in GNB include KPC, NDM, IMP, VIM, and selected OXA-type enzymes, and this review centers on these major types because of their significant clinical impact. For context and comparison with the globally distributed major carbapenemases, information is also provided on the minor, sporadically occurring *Serratia marcescens* enzyme (SME) and imipenemase/non-metallo-carbapenemase A (IMI/Nmc-A). Table 1 summarizes the most salient characteristics of these key carbapenemases in GNB.

### 3.1. Class A Serine Carbapenemases

**Figure 3:**
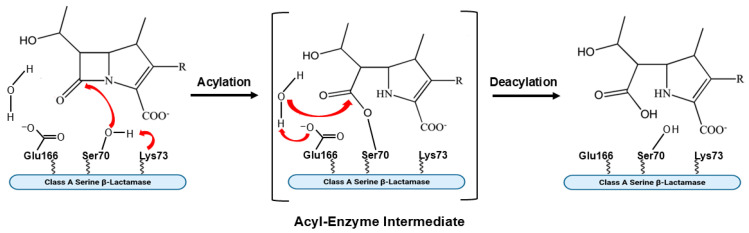
Mechanism of Carbapenem Hydrolysis by a Class A Serine β-Lactamase. Hydrolysis proceeds via a two-step acylation-deacylation reaction. In the acylation step, the nucleophilic Ser70 residue of the enzyme attacks the β-lactam carbonyl of the carbapenem. A conserved active-site Lys73 facilitates activation of Ser70 through proton transfer. In the deacylation step, a conserved Glu166 activates a catalytic water molecule that hydrolyzes the acyl-enzyme intermediate, regenerating the serine β-lactamase and releasing the inactivated carbapenem [71].

Ambler class A serine β-lactamases catalyze the hydrolysis of β-lactam antibiotics via a multistep mechanism. As shown in Figure 3, binding of the enzyme to its β-lactam substrate allows for nucleophilic attack by the active-site serine (Ser70) on the carbonyl group of the β-lactam, producing a high-energy acylation intermediate that subsequently transitions into a lower-energy covalent acyl enzyme [71]. A strategically positioned water molecule catalytically attacks the covalent complex and leads to formation of a deacylation intermediate, with hydrolysis of the bond between the β-lactam carbonyl and the serine oxygen. Deacylation results in regeneration of the nucleophilic serine in the active site of the β-lactamase and inactivation of the β-lactam antibiotic (Figure 3) [71]. The main type of carbapenem-hydrolyzing class A serine β-lactamase in Enterobacterales is KPC, with minor contributions of SME and IMI/Nmc-A, as well as other infrequently reported types not discussed here, to the clinical burden resulting from carbapenem resistance among GNB [41,43].

#### 3.1.1. SME and IMI/Nmc-A

Until recently, SME-type carbapenemases have been identified exclusively in isolates of *S. marcescens* [41], an opportunistic Gram-negative pathogen that can cause urinary tract infections, pneumonia, endocarditis, and wound infections. Five subtypes or genetic variants of SME (i.e., SME-1–5) have been reported in *S. marcescens*, with variants deviating from the originally described SME-1 by single amino acid substitutions [43]. Initially isolated from two imipenem-resistant *S. marcescens* strains in England in 1982 [72], the SME-1 β-lactamase shares an evolutionary origin with Nmc-A from *Enterobacter cloacae* NOR-1 based on amino acid sequence identity [73]. Genetic variants of SME-1 have been reported sporadically from *S. marcescens* isolates recovered in geographically diverse locations, including the UK, USA, Argentina, Switzerland, Canada, France, Brazil, and New Zealand [43,74,75,76], and typically are limited to small nosocomial outbreaks or clusters of patients [76]. Chromosomally encoded SMEs distinctively exhibit resistance to penicillins, early-generation cephalosporins, aztreonam, and carbapenems, but remain susceptible to extended-spectrum cephalosporins (e.g., ceftazidime) [45]. Recently, a novel *bla*_SME-6_ variant was detected in *Serratia ureilytica* strain X47 isolated from the sputum of a hospitalized patient in Germany [44]. SME-6 differed from SME-2 by two amino acid substitutions (G117R and G147E) and displayed a temperature-dependent resistance phenotype defined by high resistance to both imipenem and meropenem at 30 °C but carbapenem susceptibility at 37 °C.

The IMI and Nmc-A serine β-lactamases constitute a closely related group of uncommon class A carbapenemases, with Nmc-A sharing 97% amino acid sequence identity with IMI-1 [77]. IMI/Nmc-A enzymes have been identified typically in association with species of the *Enterobacter cloacae* complex [41,43], although rare reports have described IMI-2-carbapenemase–producing clinical isolates of *Escherichia coli* [78] and *Klebsiella variicola* [79]. The *E. cloacae* complex comprises diverse, opportunistic bacteria (namely, *E. cloacae*, *E. asburiae*, *E. hormaechei*, *E. kobei*, *E. ludwigii*, and *E. nimipressuralis*) associated with nosocomial infections such as pneumonia, sepsis, and urinary tract infections [80]. To date, Nmc-A and a limited number of IMI variants (currently totaling 24) have been identified. The hydrolytic activity of IMI/Nmc-A β-lactamases is similar to that of SMEs, conferring resistance to penicillins, early-generation cephalosporins, and carbapenems, but not to broad-spectrum cephalosporins [77]. While *bla*_NMC-A_-type carbapenemase genes have been found on *Enterobacter* chromosomes, the highly homologous *bla*_IMI_ variants display a more versatile genetic context, occurring on either chromosomes or plasmids [81]. *E. cloacae* complex species harbor *bla*_NMC-A_, *bla*_IMI-1_, and *bla*_IMI-9_ chromosomally integrated on Xer recombinase-dependent integrative mobile elements called EcloIMEX-like elements [81,82]. Other IMI variants, e.g., *bla*_IMI-2_, *bla*_IMI-5_, and *bla*_IMI-6_, have been identified on extra-chromosomal IncFII-type plasmids, allowing for enhanced *bla*_IMI_ mobilization and potentially rapid spread of carbapenem resistance to other bacterial genera [81,83,84].

Reports documenting the identification of IMI variants and NMC-A in the literature, although infrequent and sporadic, indicate a broad geographic distribution, with cases reported in such countries as Singapore, China, French Polynesia, Japan, the UK, the Czech Republic, Canada, Austria, Spain, and Costa Rica [43,85]. The clinical significance of IMI-producing *E*. *cloacae* complex species has recently gained increased attention, driven by reports documenting clusters of nosocomial infections and a hospital-wide outbreak likely originating from community-associated transmission [86,87]. IMI-type carbapenemase genes also have been detected in environmental compartments, particularly in aquatic ecosystems [88,89,90,91], suggesting that the environment may serve as an important reservoir for the persistence and transmission of IMI carbapenemases. Probable transmission of IMI-2–producing *E. asburiae* (IMI-2-Easb) from a river environment to human microbiota was documented in a case in which a patient developed bacteremia caused by IMI-2-Easb after an accidental near-drowning event [92].

#### 3.1.2. KPC

KPC enzymes represent the most concerning class A serine carbapenemases because of their global prevalence, efficient mobilization via self-conjugative plasmids or transposable elements, and frequent association with *K. pneumoniae*, a significant cause of pneumonia, UTIs, and bloodstream infections, particularly among immuno-compromised patients in healthcare settings [41,42]. In 2001, the first carbapenem-hydrolyzing β-lactamase (KPC-1, redesignated as KPC-2) was purified and functionally characterized from a clinical isolate of *K. pneumoniae* collected at a North Carolina (USA) hospital [46,93]. Comparative sequence analysis identified KPC-2 as a genetically distinct and novel carbapenemase exhibiting the highest amino acid sequence identity (45%) with SME-1 from *S. marcescens* S6, followed by Nmc-A (44%) and IMI-1 (43%) [46]. KPC-2 exhibited a wide substrate spectrum, hydrolyzing penicillins, oxyimino-cephalosporins, monobactams (namely, aztreonam), and carbapenems, but having the highest affinity for meropenem (K_m_ of 12 μM) [46]. The traditional β-lactamase inhibitors clavulanic acid, tazobactam, and sulbactam are ineffective against KPC-2 [47,48].

KPC variants are defined by mutations, including point substitutions, insertions, or deletions, that alter the amino acid sequence of the carbapenemase relative to the canonical forms, KPC-2 and KPC-3 [94]. As of 2 April 2026, a total of 290 distinct *bla*_KPC_ alleles have been recorded in the NCBI database (https://www.ncbi.nlm.nih.gov/pathogens/refgene/#gene_family:blaKPC, accessed on 2 April 2026). Based on recent reports on global KPC epidemiology, KPC-2 and KPC-3 continue to be the most prevalent variants worldwide [95,96], indicating that KPC genetic diversity is dominated by a few variants. While *K. pneumoniae* is the predominant host for KPC-type carbapenemases, KPC variants also have been identified in other GNB, including *E. coli*, *E. cloacae*, *Citrobacter* spp., *Klebsiella oxytoca*, *S. marcescens*, *Klebsiella aerogenes*, *Proteus* spp., *Providencia* spp., *Morganella* spp., *Raoultella* spp., as well as non-fermentative Gram-negative bacilli such as *P. aeruginosa* and *A. baumannii* [95]. The rapid diversification of bacterial hosts harboring *bla*_KPC_ since its original discovery in a *K. pneumoniae* isolate in 1996 is attributed to localization of the KPC gene on multiple plasmid types. In a study scrutinizing 435 *bla*_KPC_-carrying plasmid sequences in the NCBI database, Brandt et al. [97] found that the incompatibility plasmid group IncN was the most prevalent, followed by IncFII, IncR, and IncA/C2. The KPC gene was located on variants of the Tn3-based transposon, Tn*4401*, in about half of all representative plasmids, underscoring the importance of Tn-mediated transposition in disseminating *bla*_KPC_ across diverse plasmid types [97].

KPCs have disseminated both regionally and worldwide [49,98,99], with endemicity reported in countries including the USA, Argentina, Greece, Italy, and China [49]. Analysis of 687 carbapenem-resistant clinical isolates, predominantly recovered from blood and urine samples and submitted to the EURECA collection from 41 hospitals across nine Southern European countries between 2016 and 2018, demonstrated that *bla*_KPC-like_ genes were the most prevalent carbapenemase-encoding determinants (46%) [100]. The widespread epidemiologic success of KPC is largely the outcome of extensive clonal dissemination. Most CRKP isolates in the EURECA study belonged to certain clonal lineages of *K. pneumoniae*, particularly the high-risk clones ST258/512, ST101, ST11, and ST307 [100].

Historically, *K. pneumoniae* KPC has been the predominant carbapenemase detected in CRE in medical facilities across the USA, although prevalence can vary substantially by geographic location [98,101]. In a recent surveillance study, 62.4% of carbapenem-nonsusceptible Enterobacterales clinical isolates collected in USA hospitals from 2016 to 2020 contained *bla*_KPC_, with *bla*_KPC-2_ and *bla*_KPC-3_ constituting the most common variants identified [101]. While KPC-positive CRE cases in New York City (USA) remained relatively stable from 2019 to 2024, citywide incidences of the metallo-β-lactamase NDM (discussed in greater detail in Section 3.2.2 below) increased annually among CRE clinical isolates and surpassed KPC as the most frequently reported carbapenemase in 2024 [102].

Most recently, CRKP clinical isolates coproducing KPC and NDM carbapenemases have been identified in Chile [103], China [104,105,106,107,108,109], Greece [110], Italy [111], Egypt [112], Brazil [113], and Argentina [114,115]. Simultaneous co-production of three major carbapenemases (KPC, NDM, and OXA) in *K. pneumoniae* has been reported in Turkey [116] and India [117], and in both cases, the strains were recovered from ICU patients. The coexistence of multiple carbapenemases in serious nosocomial bacterial pathogens is an alarming, emerging trend that threatens to lead to higher treatment failures and increased mortality rates due to reduced efficacy of available therapeutic options. KPC and NDM exhibit complementary carbapenem-hydrolyzing activity; thus, co-carriage of these enzymes effectively nullifies inhibition by certain class-targeted BLIs, e.g., serine β-lactamase inhibitors. Even more concerning, the presence of two or more carbapenemases from distinct families within a single bacterium reflects high genetic plasticity and evolutionary progression toward extreme antimicrobial resistance.

### 3.2. Class B Metallo-β-Lactamases with Carbapenem-Hydrolyzing Activity

Ambler class B carbapenemases are MBLs that are structurally distinguished by an essential zinc ion bound directly to the active site and serving as a cofactor to activate a water molecule for β-lactam hydrolysis [39]. Typically, B1 and B3 subclasses of these metalloenzymes require two zinc ions in their active site coordinated by histidine, aspartic acid, and cysteine residues, while the B2 subclass requires one zinc ion to be active. Functionally, MBLs can hydrolyze penicillins, cephalosporins, and carbapenems, but, in contrast to serine β-lactamases, they have poor affinity for monobactams like aztreonam [39]. Additionally, MBLs are not inhibited by clavulanic acid or tazobactam but are susceptible to inhibition by such metal ion chelators as ethylenediaminetetraacetic acid (EDTA), dipicolinic acid, or 1,10-*o*-phenanthroline [118]. The most encountered metallo-carbapenemases in the clinical setting are IMP, NDM, and VIM of the B1 subclass.

#### 3.2.1. IMP

IMP was first described in Japan in 1991, where it was initially isolated from a clinical imipenem-resistant *P. aeruginosa* strain during a period when imipenem was being widely used for the chemotherapy of diseases caused by GNB [50]. This IMP enzyme was mediated by a conjugative plasmid and exhibited a broad substrate profile, conferring resistance to imipenem, oxyiminocephalosporins, 7-methoxycephalosporins, and penicillins, while remaining susceptible to aztreonam, consistent with the functional behavior of other MBLs [50]. Soon thereafter, this same IMP-1 was identified on the chromosome of a clinical carbapenem-resistant *S. marcescens* isolate [119] and, in a separate *S. marcescens* strain, within an integron-like element carried on a large transferable plasmid [120]. A recent analysis of the genetic context of *bla*_IMP_ genes demonstrated that these resistance determinants are predominantly associated with mobile gene cassettes inserted in plasmid- or chromosome-borne class I integrons [51].

As of April 2026, the IMP family includes 107 genetically distinct variants (https://www.ncbi.nlm.nih.gov/pathogens/refgene/#gene_family:blaIMP, accessed on 2 April 2026). Evidence indicates that certain IMP variants possess amino acid substitutions relative to their nearest IMP homolog, which are associated with increased catalytic activity toward carbapenems in these more newly evolved enzymes [121,122,123]. For example, two novel IMP variants, IMP-43 and IMP-44, were identified in MDR *P. aeruginosa* isolates obtained from medical facilities in Japan. The sequence of IMP-43 had one amino acid substitution (V67F) compared to IMP-7, and IMP-44 had two amino acid substitutions (V67F and F87S) compared with IMP-11 [121]. While IMP-43 exhibited greater catalytic efficiency against doripenem, meropenem, and imipenem than IMP-7, IMP-44 demonstrated increased catalytic activity against all carbapenems tested compared with both IMP-11 and IMP-43, indicating that the V67F and F87S substitutions together contribute to enhanced hydrolytic efficiency [121]. Cheng et al. [123] found that the increased resistance toward carbapenems associated with clinically derived IMP-1-like variants harboring V67F or S262G substitutions was likely driven by exposure to structurally different β-lactam drugs, primarily meropenem and ertapenem, and not by zinc(II) scarcity.

IMP variants show noteworthy differences in molecular epidemiology and predominant bacterial hosts compared to KPC. Genetically diverse IMP variants demonstrate limited global dominance, with the highest historical prevalence in East Asia. This MBL family of carbapenemases is frequently identified in non-fermenters such as *P. aeruginosa*, but are also found in Enterobacterales due to horizontal gene transfer-mediated diversification. In general, IMPs constitute the most prevalent MBL-type carbapenemases in Asia and the South Pacific (e.g., Japan, China, Taiwan, and Australia), where their prevalence is considered endemic [52,53,124]. However, IMP-producing organisms exhibit a broad geographical distribution, with sporadic and localized outbreaks reported in medical settings in such countries as Italy [125], Spain [126], Brazil [127,128], and Egypt [129]. Individual IMP variants tend to be regionally restricted. For example, the key *bla*_IMP_ subtypes present in Japan are *bla*_IMP-1_ and *bla*_IMP-6_ [60,124]. The predominant MBL identified in Australia is the variant IMP-4, and *E. cloacae* has supplanted *S. marcescens* as the principal species associated with *bla*_IMP_ carriage in that country [53,130]. The early emergence of *bla*_IMP-1_ and *bla*_IMP-4_ resulted in their global dominance and endemicity, along with variants *bla*_IMP-7_, *bla*_IMP-8_, and *bla*_IMP-13_ [53]. Other *bla*_IMP_ variants exhibit a more confined pattern of geographical distribution, with *bla*_IMP-26_ and *bla*_IMP-27_ becoming regionally endemic in Southeast Asia and North America (specifically the USA), respectively [53]. Additionally, IMP variants are associated with specific bacterial hosts. The most common species harboring *bla*_IMP-1_, *bla*_IMP-4_, and *bla*_IMP-6_ are among the Enterobacterales and include *Enterobacter hormaechei*, *E. cloacae*, *K. pneumoniae*, and *E.coli*; whereas, *bla*_IMP-7_, *bla*_IMP-13_, and *bla*_IMP-26_ are predominantly identified in *P. aeruginosa* [53].

#### 3.2.2. NDM

The Indian subcontinent represents the epicenter for the emergence and dissemination of NDM-producing Enterobacterales [54,131]. NDM-1 was first identified in 2009 in *K. pneumoniae* and *E. coli* isolates recovered from a Swedish patient who had been hospitalized in New Delhi, India [55]. NDM-1 is structurally unique from other MBLs, showing the highest amino acid sequence identity at 32.4% to VIM-1/VIM-2 and efficiently hydrolyzes all β-lactams, except aztreonam [55]. The *bla*_NDM-1_ gene has been identified on diverse, highly mobile plasmids of both narrow host range (e.g., IncF types) and broad host range (e.g., IncA/C types), facilitating the widespread dissemination of NDM-1 among Enterobacterales, as well as *Acinetobacter* and *Pseudomonas* species [132]. Moreover, *bla*_NDM-1_ plasmids commonly coharbor other antimicrobial resistance determinants, including CMY-type plasmid-mediated AmpC β-lactamases, CTX-M–type ESBL genes (especially *bla*_CTX-M-15_), other types of carbapenemase genes (*bla*_OXA_ and *bla*_KPC_), and genes encoding enzymes that confer broad-spectrum resistance to aminoglycosides (16S RNA methylase genes) and quinolones (*qnr*, *aac(6′)-Ib-cr*, *qepA*) [132,133]. The co-resistance phenotypes observed in NDM-producing *K. pneumoniae* and *E. coli* are particularly troubling because these pathogens are major causes of nosocomial and community-acquired infections, respectively [131]. Accordingly, NDM producers are associated with a wide range of infections, including UTIs, bacteremia, pneumonia, and wound infections.

NDM enzymes exhibit substantial variant diversity and a propensity to evolve enhanced carbapenem-hydrolyzing activity under selective pressure. The number of NDM variants registered in the NCBI database currently includes 96 (https://www.ncbi.nlm.nih.gov/pathogens/refgene/#gene_family:blaNDM, accessed on 2 April 2026), with NDM-1, NDM-4, NDM-5, and NDM-7 representing the most frequently reported variants. NDM-4, NDM-5, and NDM-7 exhibit increased hydrolytic activity toward carbapenems and in certain cases, several cephalosporins compared to that of NDM-1 [134,135,136,137]. In the case of NDM-4, this variant differs from the canonical NDM-1 by a single amino acid substitution at Met154 (M154L) [134]. The Leu for Met substitution is the most common sequence change found in all naturally-occurring clinical NDM variants [138]. NDM-4 exhibits increased carbapenemase activity relative to NDM-1, with higher catalytic efficiencies for the hydrolysis of both imipenem and meropenem [134]. The dinuclear zinc cluster in the structure of NDMs is critical for catalysis of β-lactam substrate hydrolysis. The two zinc centers, connected by a single-atom bridge, form a highly effective catalyst with two active sites: one functioning as a Lewis acid and the other as a Brønsted base [139]. The proximity of the M154L substitution to the dinuclear zinc cluster is significant and likely illustrates the evolution of NDMs toward modulating the structural environment of the active site for improved carbapenem catalysis [138]. Research by Stewart et al. [138] demonstrated that NDM-4 binds zinc(II) with greater affinity than NDM-1, resulting in enhanced catalytic efficiency against carbapenems, and suggests that certain NDM variants have evolved to overcome the dual selective pressures of β-lactam exposure and zinc(II) scarcity during infection.

Since the initial description of NDM-1 in 2009, *bla*_NDM-1_ and its variants have undergone rapid and widespread interspecies dissemination to a greater extent compared to other MBLs like IMP and VIM [140]. In a global surveillance study conducted from 2012 to 2014, Kazmierczak et al. [52] reported that among GNB isolates collected from patients in 40 countries, 44.2% of MBL-positive Enterobacterales harbored *bla*_NDM_, with NDM-1 being one of the most prevalently identified variants of the MBL types. NDM was the predominant MBL type identified across the regions of Africa, Asia, Europe, Latin America, and the Middle East [36,140]. Moreover, the incidence of NDM-producing CRE clinical isolates is increasing in the USA, where it was once relatively uncommon. Analysis of data collected by the CDC’s Antimicrobial Resistance Laboratory Network revealed that the age-adjusted incidence of NDM-CRE surged by 461% (incidence rate ratio [IRR], 5.61 [CI, 4.96–6.36]) between 2019 and 2023, with NDM identified in 27%, 24%, and 6% of carbapenem-resistant *E. coli*, *Klebsiella* spp., and *Enterobacter* spp., respectively [33]. In a recent analysis of reported population-based neonatal CRKP infections, Hu et al. [141] found that NDM was the most common carbapenemase type (64.3%) identified in clinical isolates recovered in 14 countries. The pooled mortality of hospitalized neonates with CRKP infections was 22.9% [141]. While the dominant bacterial hosts for NDM genes are *K. pneumoniae* and *E. coli*, *bla*_NDM_ is also associated with such important opportunistic pathogens as *A*. *baumannii* and *P*. *aeruginosa* [42]. NDM-1-positive bacteria also have been detected in environmental sources such as seepage and tap water, river water, sewage treatment plants, and hospital effluents from India [142,143,144]. The persistence of NDM in these environments suggests that community exposure to this clinically significant resistance determinant is likely to increase over time, impacting the health of more individuals and elevating dissemination.

#### 3.2.3. VIM

VIM enzymes represent another prevalent family of integron-associated zinc-dependent MBLs that are highly divergent at the sequence level from other class B metalloenzymes. Compared to NDM, VIM enzymes display integron-mediated diversification and a lower global prevalence. VIM-1 and VIM-2 were originally identified in the 1990s in carbapenem-resistant *P. aeruginosa* clinical isolates obtained from hospitalized patients in Italy and France, respectively [56,57]. In both cases, the *bla*_VIM-1_ and *bla*_VIM-2_ gene cassettes were inserted into a class 1 integron. VIM MBLs display a broad substrate hydrolysis profile, which includes penicillins, cephalosporins, cephamycins, oxacephamycins, and carbapenems, but not monobactams [56,57]. The VIM family currently comprises 94 variants (https://www.ncbi.nlm.nih.gov/pathogens/refgene/#gene_family:blaVIM, accessed on 2 April 2026). The most frequently detected *bla*_VIM_ variant among clinical isolates of MBL-producing Enterobacterales (primarily *K. pneumoniae*, *E. coli*, and *Enterobacter* spp.) is *bla*_VIM-1_ [36,145,146].

VIM epidemiology is characterized by regional endemicity. Mediterranean Europe (particularly Italy, Greece, and Spain) continues to be the major reservoir for VIM producers, which are concentrated in this geographical region; however, bacterial species harboring *bla*_VIM_ have been detected worldwide [36,145,147]. In a recent pangenomic study, Zhai et al. [58] investigated the distribution of MBL-encoding genes in *P. aeruginosa* strains isolated worldwide from predominantly human sources. The WHO has designated *P. aeruginosa* as a high-priority pathogen due to its increasing carbapenem resistance, which severely limits antimicrobial chemotherapeutic options [1,148]. Of the 21,788 global genomes analyzed, approximately 12% of strains contained 4014 *bla*_MBL_ genes, of which 51.2% were *bla*_VIM_, 24.1% *bla*_IMP_, and 23.4% *bla*_NDM_ [58]. The *bla*_VIM-2_ gene was the most common VIM variant identified among MBL-producing *P. aeruginosa* isolates, accounting for 73.2% [58]. *P. aeruginosa* isolates harboring *bla*_VIM_ genes have been associated with nosocomial outbreaks in different parts of the world, including prolonged outbreaks in a French surgical ICU [149]; ocular and systemic infections [150] as well as outbreaks in academic healthcare systems and long-term acute care hospitals in the USA [151,152]; ICUs in Belgium [153,154] and Austria [155]; tertiary care hospitals in Sweden [156,157]; and a nationwide, inter-institutional outbreak in the Netherlands [158]. Less common bacterial hosts of VIM-type MBLs include *Achromobacter xylosoxidans*, *A. baumannii*, *S. marcescens*, and *Citrobacter freundii* [147]. Recent epidemiology studies indicate that hospital-acquired infections are increasingly attributable to carbapenemase-producing *C. freundii* [159,160], with VIM-1 ranking as the second most frequently reported carbapenemase associated with this species after KPC-2 [161].

### 3.3. Carbapenem-Hydrolyzing Class D Serine β-Lactamases (Selected OXA Families)

The OXA family of class D serine β-lactamases is extremely diverse, with >1300 distinct OXA sequences recorded in the NCBI database (currently 1381 total OXA variants, https://www.ncbi.nlm.nih.gov/pathogens/refgene/#gene_family:blaOXA [accessed on 2 April 2026]). OXA β-lactamases are defined by their strong preferential catalytic activity toward oxacillin and other semisynthetic penicillins (e.g., methicillin, cloxacillin) and a reduced capacity to hydrolyze benzylpenicillin, a kinetic profile that distinguishes these enzymes from class A serine β-lactamases [59]. Additionally, OXA enzymes display weak activity against extended-spectrum cephalosporins (e.g., ceftazidime, cefepime) [59]. A subset of OXA-family β-lactamase variants exhibit documented carbapenemase activity, which is typically weaker from that of KPC and MBLs. These carbapenem-hydrolyzing OXAs have emerged as increasingly important contributors to clinical carbapenem resistance in *A. baumannii* and certain nosocomial pathogens of the Enterobacterales [14,59]. At present, the NCBI database lists 17 genetically distinct OXA families of carbapenem-hydrolyzing class D β-lactamases, organized according to evolutionary relatedness (amino acid sequence identity and phylogeny) to the prototype enzyme. Here, we focus on the five predominant OXA carbapenemase families in terms of clinical significance and frequency of reporting: OXA-23-like, OXA-24/40-like, OXA-51-like, OXA-58-like, and OXA-48-like β-lactamases. Closely related variants within a family hydrolyze carbapenems to some degree but may differ in kinetic efficiency and substrate spectra.

Carbapenem resistance in *A*. *baumannii* is primarily mediated by OXA carbapenemases of the OXA-23, OXA-24/40, OXA-51, and OXA-58 families, which are characteristically *Acinetobacter*-associated, in contrast to OXA-48 variants. The first carbapenem-hydrolyzing OXA β-lactamase, OXA-23, was described in 1993 for an imipenem-resistant strain of *A. baumannii* isolated from the blood of a patient hospitalized in the UK [60,61]. The OXA-23-like family currently includes 55 distinct allelic variants that share high sequence identity (>95%) to OXA-23. These variants have been reported largely in various *Acinetobacter* species and much less frequently in *K. pneumoniae* and *Proteus mirabilis* [59,162,163,164,165]. Notably, OXA-23-like carbapenemases are the most globally prevalent acquired determinants in *A. baumannii*, and *bla*_OXA-23_-harboring *Acinetobacter* isolates are globally disseminated [62,166]. OXA-23-like genes have been identified in both chromosomal and plasmid contexts, but, unlike many class D oxacillinases, these OXA-type carbapenemases are not integrated into integron-mediated gene cassettes [59,167]. The carbapenem hydrolytic activities of OXA-23 and related variants are typically weak compared with class A (KPC) and class B (MBL) carbapenemases but sufficient to confer clinical resistance [59,168]. The catalytic profile of OXA-23 is characterized by a much higher turnover rate (*k*_cat_ value) for imipenem (0.35 ± 0.01 s^−1^) than for meropenem (0.068 ± 0.001 s^−1^), doripenem (0.036 ± 0.001 s^−1^), and ertapenem (0.021 ± 0.001 s^−1^) [63]. Clinical resistance of OXA-23-producing *A. baumannii* to carbapenems is enhanced by carbapenemase expression levels combined with decreased outer membrane permeability and overexpression of drug efflux pumps [59,63,169,170].

OXA-24, subsequently renamed OXA-40, was first identified in the chromosome of a carbapenem-resistant clinical strain of *A. baumannii*, which was attributed to a prolonged hospital outbreak in Spain in 1997 [64]. The OXA-24/40 enzyme exhibited a moderately efficient rate of hydrolysis for imipenem (relative *V*_max_/*K_m_*, 13) and meropenem (relative *V*_max_/*K_m_*, 6) [64]. While OXA-23 and OXA-24/40 are major contributors to carbapenem resistance in *A. baumannii* isolates, Héritier et al. [171] demonstrated that these OXA enzymes work in concert with the overexpression of the AdeABC efflux pump to achieve high levels of clinical carbapenem resistance. Genes encoding OXA-24/40-like variants occur on either chromosomes or plasmids in *Acinetobacter* spp. and less commonly in *P. aeruginosa* and *K. pneumoniae*, thus increasing the risk of horizontal gene transfer in hospital settings [59,172,173,174].

The OXA-51 family comprises the largest number of allelic variants (398 to date), distinguished by amino acid substitutions from the OXA-51 prototype. The extensive genetic diversification of OXA-51-like enzymes suggests ongoing emergence of novel forms driven by carbapenem selective pressure, with evidence that clinically prevalent substitutions in the OXA-51 prototype enhance carbapenemase activity [59,175,176]. In general, OXA-51-like β-lactamases have weak intrinsic carbapenem hydrolysis activity and are inherent, naturally occurring chromosomally encoded resistance determinants in *A. baumannii* [59,65]. Plasmids harboring *bla*_OXA-51-like_ also have been detected in *Acinetobacter nosocomialis* and in members of the Enterobacterales [177,178]. Studies suggest that intrinsic OXA-51-like variants confer little to no clinically meaningful carbapenem resistance unless the insertion sequence IS*Aba1* is located immediately upstream of the *bla*_OXA-51-like_ gene, where it likely serves as a promoter sequence driving overexpression of the *bla*_OXA-51-like_ gene in *A. baumannii* isolates [66,179,180,181]. IS*Aba1* and IS*Aba4* elements have been identified upstream of *bla*_OXA-23_ as well and are strongly associated with carbapenem resistance [66,179,180,181,182]. A recent study showed that IS*Aba1*-driven overexpression of OXA-51-like genes, together with specific active-site amino acid substitutions, was sufficient to enhance carbapenem resistance in *A. baumannii*, without the requirement for additional synergistic resistance mechanisms [176].

Since the discovery of a plasmid-encoded OXA-58 in 2003 [67], only seven additional variants in this family have been recorded in the NCBI database. Like other OXA carbapenemases, OXA-58 has a narrow-spectrum hydrolysis profile, with weak activity against carbapenems and penicillin and no measurable activity against extended-spectrum cephalosporins [67]. Plasmid mobilization is the principal driver of OXA-58 dissemination among *Acinetobacter* species, and *bla*_OXA-58_ is frequently flanked by insertion sequences (e.g., IS*Aba1*, IS*Aba2*, IS*Aba3*, or IS*18*) that provide promoter sequences enhancing gene expression [66,67,183]. Recent reports of plasmid-mediated *bla*_OXA-58_ co-carriage with *bla*_IMP_ or *bla*_NDM-1_ across diverse *Acinetobacter* and non-*Acinetobacter* species increase the risk of treatment failure in hospital settings e.g., [184,185,186,187,188,189,190].

Acquired OXA-48-like carbapenemases contribute to the global rise of carbapenem-nonsusceptible Enterobacterales. In 2001, the founding member of this family, OXA-48, was first identified in a *K. pneumoniae* clinical isolate recovered from a hospitalized patient in Turkey. Kinetic analysis of the plasmid-encoded OXA-48 demonstrated that the enzyme had a narrow-spectrum substrate profile that included penicillins, imipenem, and to a substantially lesser extent, meropenem, but not expanded-spectrum cephalosporins [68]. Although possessing weak carbapenemase activity overall, the catalytic efficiency of OXA-48 for imipenem was 10-fold higher than that of OXA-40 from *A. baumannii* and 3-fold higher than KPC-1 [68]. OXA-48 enzymes are essentially imipenemases with low-level hydrolytic activities against meropenem and ertapenem. Currently, the OXA-48 family includes OXA-48 and 22 derivatives, which show variable catalytic efficiencies for carbapenems compared to the prototype [59,191]. OXA-48 and related variants can confer high carbapenem MICs in *bla*_OXA-48_-positive *K. pneumoniae* isolates when enzyme production is coupled with outer membrane permeability defects due to porin loss or altered expression [68,192]. Deficiency of OmpK36 combined with high copy numbers of *bla*_OXA-48_-carrying plasmids synergistically contributes to elevated imipenem and meropenem MICs [193]. The *bla*_OXA-48_ gene is commonly located in Tn*1999*-like composite transposons harbored on the IncL/M-type conjugative plasmid pOXA-48a, which is mainly responsible for the widespread dissemination of *bla*_OXA-48_ in *K. pneumoniae* and other Enterobacterales [69,194].

OXA-48-like enzymes are predominantly detected in hospital-acquired *K. pneumoniae* and community-acquired *E. coli*, as well as *E. cloacae* isolates [49,70,195,196]. Epidemiology data from the SMART global surveillance program (2008 to 2014) indicate that Africa (70.0%), the Middle East (48.8%), and Europe (29.0%) represent the regions with the highest prevalence of OXA-48-like enzymes among detected carbapenemases [196]. A similar geographic distribution was reported in a more recent global surveillance study, with no *bla*_OXA-48-like_ genes detected in isolates collected in the USA between 2012 and 2017 [49]. Endemic levels of *bla*_OXA-48_-positive Enterobacterales currently exist in Turkey, North Africa, and the Middle East [70]. Notably, high percentages (88.7–90.9%) of globally surveyed Enterobacterales isolates expressing OXA-48-like enzymes co-carried additional β-lactamases, such as ESBLs (particularly CTX-M-15, CTX-M-14, and CTX-M-3), AmpC, and MBLs, capable of expanding the multidrug-resistant phenotype of isolates beyond the restricted hydrolytic profiles of OXA-48 and its variants [49,197].

## 4. Next-Generation β-Lactam/β-Lactamase Inhibitor Combinations

The rapid global dissemination of carbapenemases—particularly KPC variants, MBLs, and OXA-48-like oxacillinases—among serious nosocomial bacterial pathogens represents one of the most pressing public health challenges, as these enzymes are not effectively inhibited by classical BLIs (e.g., CLAV, SUL) and severely limit therapeutic options available to patients. The development of novel inhibitors in combination with established β-lactams, both in clinical use and currently progressing in the pipeline, marks an important advance in strengthening the therapeutic armamentarium for the treatment of MDR Gram-negative infections. Here we provide up-to-date information on recently approved and experimental BL/BLI combinations, discuss their mechanisms of action, and evaluate gaps in their microbiological and spectrum of activity, with a focus on carbapenemases (summarized in Table 2).

### 4.1. Ceftazidime-Avibactam (CAZ-AVI)

CAZ-AVI is an intravenously administered antibiotic composed of a third-generation cephalosporin, CAZ, and a novel non-β-lactam β-lactamase inhibitor, AVI [199,266]. CAZ inhibits PBPs, disrupting peptidoglycan crosslinking during cell wall biosynthesis. The broad-spectrum antibacterial activity of CAZ is protected by AVI, a synthetic diazabicyclooctane (DBO) BLI, that covalently and reversibly binds to serine β-lactamases and demonstrates potent inhibition of Ambler class A (ESBLs, KPC), chromosomal and acquired class C (AmpC), and specific class D (e.g., OXA-48-like) β-lactamases [200,201,267,268]. However, AVI lacks efficacy against Ambler class B MBLs (NDM, VIM, IMP) [201]. CAZ-AVI was approved for the treatment of complicated intra-abdominal infections (cIAIs), complicated urinary tract infections (cUTIs), and hospital-acquired bacterial pneumonia (HABP), including ventilator-associated pneumonia (VABP), based on efficacy and safety data collected from the RECLAIM [269], RECAPTURE [270], and REPROVE [271] clinical trials. In addition to its lack of activity against MBLs, a major limitation of CAZ-AVI is the emergence of clinical resistance.

CAZ-AVI resistance emerged relatively rapidly following its introduction in clinical practice, with occurrences of nonsusceptibility now being reported across Enterobacterales and *P. aeruginosa* isolates [202,203]. A recent systematic review and meta-analysis showed that the proportion of CAZ-AVI resistance increased significantly among GNB isolates from 5.6% (95% CI 4.1–7.6) in 2015–2020 to 13.2% (95% CI 11.4–15.2) in 2021–2024, and CAZ-AVI resistance rates were the highest in Asia (19.3%), followed by Africa (13.6%), Europe (11%), South America (6.1%), and North America (5.3%) [203]. Because AVI has no inhibitory effect on MBLs, the production of MBLs in KPC-positive *K. pneumoniae* strains is a predominant intrinsic mechanism of CAZ-AVI resistance [272]. Huang et al. [104] demonstrated that a KPC-2–producing *K. pneumoniae* strain developed resistance to CAZ-AVI during therapeutic treatment by acquiring a *bla*_NDM-5_-carrying plasmid.

A primary driver of treatment-emergent resistance is the evolution of point mutations, insertions, and deletions in *bla*_KPC-2_ and *bla*_KPC-3_ genes, leading to the expression of novel KPC variants resistant to CAZ-AVI [96,272]. These resistance-related mutations have been described to occur in various “hot spots” in the KPC enzyme, namely the Ω-loop (residues 164–179) bordering the catalytic pocket, loop 237–243, and loop 266–275 [96]. Mutations in the Ω-loop structural region of KPCs can enhance CAZ affinity and reduce AVI binding [273]. Among CAZ-AVI–resistant KPC variants identified to date, the most frequently reported clinical variants are KPC-31 and KPC-33 containing the D179Y +/− H274Y mutation(s), and KPC-35 containing the L169P substitution [274,275]. Other recently described CAZ-AVI resistant variants of KPC-2 include KPC-179 (A133T substitution + 183S insertion) [276], KPC-190 (D179Y + A243V substitutions) [277], and KPC-228 (del_167–170 ELNS) with a deletion of four amino acids in the Ω-loop [278]. While resistance arises predominantly from selective pressure of prior CAZ-AVI exposure, a rare case of de novo CAZ-AVI resistance has been reported [279], underscoring the therapeutic challenge of managing infections caused by carbapenemase-producing GNB. Additionally, outer membrane permeability defects, overexpression of efflux pumps, and mutations in the PBP3-encoding gene *ftsI* [202,280] often coexist with KPC variants and contribute to increased MICs for CAZ-AVI.

### 4.2. Meropenem-Vaborbactam (MER-VAB)

MER-VAB, administered intravenously, combines the carbapenem MER with a novel cyclic, boronic acid-based, non-β-lactam BLI (VAB) [204,205]. VAB, which enters the periplasm of GNB through the major outer-membrane porins OmpK35 and OmpK36, inhibits class A KPCs and class C β-lactamases, thereby protecting MER from enzymatic hydrolysis, but it is not active against class B MBLs or class D (OXA-48-like) carbapenemases [208]. MER-VAB targets and exhibits potent in vitro activity against clinical isolates of KPC-producing Enterobacterales [205,206]. It was the first FDA-approved carbapenem/BLI combination therapeutic with activity against CRE for the treatment of adults with cUTIs, including acute pyelonephritis (AP) [281]. In 2018, the European Medicines Agency (EMA) expanded MER-VAB indications to include cUTI, cIAI, and HABP or VABP [282]. A Phase 3 randomized controlled trial (TANGO II) found that monotherapy with MER-VAB for patients with a CRE infection (including BSI, cUTI/AP, cIAI, and HABP/VABP) was associated with an increase in clinical and microbiologic cure, as well as decreased mortality, compared with the best available therapy [207].

### 4.3. Imipenem-Cilastatin-Relebactam (IMI-REL)

IMI-REL is an intravenously administered combination of the carbapenem IMI, the renal dehydropeptidase-I inhibitor cilastatin, and a novel bicyclic DBO β-lactamase inhibitor, REL [209]. Cilastatin prevents the renal metabolism of IMI by competitively inhibiting dehydropeptidase-1 along the renal tubules, although it has no antibacterial activity itself [283,284]. The addition of REL significantly potentiates the activity of IMI against most Enterobacterales species and *P. aeruginosa*, but not against *A. baumannii* [210]. IMI-REL has demonstrated efficacy against CRE harboring class A and class C β-lactamases, including KPC serine carbapenemases, but exhibits little-to-no activity against OXA-48-producing CRE and no activity against MBL (NDM, IMP, VIM) producers [211,212,285,286,287]. IMI-REL is approved for the treatment of cUTIs, cIAIs, HABP, and VABP. Resistance to IMI-REL has been documented among KPC-producing *K. pneumoniae* isolates due to porin (OmpK35 and OmpK36) loss of function (decreased permeability), KPC allele mutations, and increased *bla*_KPC_ copy number [213,214].

### 4.4. Sulbactam-Durlobactam (SUL-DUR)

SUL-DUR is an intravenous BL/BLI combination approved by the USA FDA in 2023 for treatment of adult patients with HABP/VABP caused by susceptible isolates of *Acinetobacter baumannii*-*calcoaceticus* complex (ABC) [215]. *A. baumannii* is a globally prevalent, difficult-to-treat nosocomial pathogen with a strong propensity for multidrug resistance, including carbapenem resistance, and has been designated a critical-priority target by the WHO for new antimicrobial development [1,288]. SUL, a penicillanic acid, is a β-lactam with intrinsic antibacterial activity against *Acinetobacter* species and an established class A serine β-lactamase inhibitor [289,290]. SUL inhibits *Acinetobacter* transpeptidases PBP1 and PBP3, which are essential enzymes in cell wall biosynthesis [291]. Various β-lactamases, such as class A TEM-1 and class D OXAs, acquired or overexpressed by contemporary *Acinetobacter* isolates, have degraded the bactericidal functionality of SUL [290,292,293]. DUR is a novel DBO BLI with a broad-spectrum of activity against clinically relevant class A, C, and D serine β-lactamases, including OXA-type carbapenemases (e.g., OXA-24) which are prevalent in carbapenem-resistant ABC complex species, and as a result, protects SUL from hydrolysis by ABC-produced β-lactamases [218,219]. Like other DBO β-lactamase inhibitors, DUR does not inhibit class B MBLs [218]. Although less prevalent than acquired *bla*_OXA_ carbapenemase genes, the dissemination of *bla*_NDM_, *bla*_IMP_, and *bla*_VIM_ among healthcare-associated *Acinetobacter* isolates has increased SUL-DUR non-susceptibility [288].

### 4.5. Cefepime-Enmetazobactam (CFP-ENM)

CFP-ENM combines a broad-spectrum 4th-generation cephalosporin (CFP) with a novel penicillanic acid sulfone β-lactamase inhibitor (ENM) structurally related to tazobactam and with potent activity against ESBL-producing Enterobacterales [220,221]. Both CFP and ENM are zwitterionic, a property that enhances their potency and facilitates penetration of the bacterial cell wall [222,294]. ENM effectively inactivates CTX-M, TEM, and SHV ESBLs, as well as other class A β-lactamases, thus protecting CFP from hydrolysis by class A ESBLs and restoring CFP’s bactericidal activity in vitro and in vivo against Enterobacterales producing ESBLs [222]. Because carbapenems are resistant to ESBL-mediated hydrolysis, this class of β-lactams has generally been used to treat infections due to ESBL-producing bacteria [223]. Therefore, CFP-ENM may serve as a potential carbapenem-sparing alternative to the treatment of infections caused by ESBL producers [220,223]. CFP-ENM was approved by the USA FDA in February 2024 for treatment of adult patients with cUTI including AP, caused by susceptible strains of *E. coli*, *K. pneumoniae*, *P. aeruginosa*, *Proteus mirabilis*, and *E. cloacae* complex [220,221]. In March 2024, the EMA expanded the approved indications for CFP-ENM to cover cUTIs (including AP), HABP/VABP, and bacteremia [221].

While clinical data supporting the use of CFP-ENM to treat carbapenemase producers does not currently exist, in vitro studies have shown the potency of CFP-ENM against CRE isolates producing OXA-48-like β-lactamases [220,224,243,295]. In a large comparative in vitro analysis of different BL/BLIs, Bonnin et al. [224] reported that CFP-ENM and CAZ-AVI displayed similar rates of susceptibility (96.7% vs. 99.5%, respectively) against OXA-48 producers. However, CFP-ENM showed less efficient activity against KPC producers, with different studies reporting CFP-ENM susceptibility rates of 63.3% [224] and 54% [295]. Although the use of CFP-ENM in infections caused by ESBL/OXA-48-like co-producers appears to be promising, in vivo studies are needed to confirm whether this is a valuable therapeutic approach to infections caused by OXA-48-positive GNB.

### 4.6. Aztreonam-Avibactam (ATM-AVI)

The intravenous ATM-AVI co-formulation comprises the monobactam ATM, which contains a unique monocyclic β-lactam ring, and the novel non-β-lactam, DBO β-lactamase inhibitor AVI. ATM selectively targets PBP3 with high affinity to disrupt cell wall synthesis [296]. In contrast to other β-lactam antibiotic classes, the unique monocyclic β-lactam ring of ATM is inherently stable to class B MBL hydrolysis; however, serine β-lactamases, like ESBLs and AmpC, can inhibit the antibacterial activity of ATM [297,298]. AVI potently inactivates class A and class D OXA-48-like carbapenemases, as well as a wide range of class A ESBLs and AmpC β-lactamases, but is ineffective against MBLs [201]. Co-administration of ATM and AVI addresses an important spectrum gap by permitting microbiologically active coverage of MBL-producing isolates of Enterobacterales, which often co-produce serine β-lactamases [226].

Numerous susceptibility studies have demonstrated the robust antimicrobial activity of ATM-AVI against global isolates of Enterobacterales exhibiting carbapenem nonsusceptibility and/or carbapenemase production, including MBLs [197,227,228,229,299]. Sader et al. [227] showed that ATM-AVI was highly active against all CRE isolates tested, including clinical strains producing KPC, OXA-48-like, and MBL carbapenemases that were collected from the USA and 19 other countries. When compared to susceptible Enterobacterales isolates, the in vitro activities of ATM-AVI and ATM alone were less potent against *P. aeruginosa* (MIC_90_, 32 μg/mL vs. MIC_90_, 0.12 μg/mL), and the efficacy of ATM was not enhanced by the addition of AVI, suggesting that nonenzymatic mechanisms contribute to ATM resistance in *P. aeruginosa* [228]. ATM alone or in combination with AVI also had no in vitro activity against *A. baumannii* isolates [299], pointing to an important gap in ATM-AVI’s microbiological coverage. Recent investigations demonstrated that ATM-AVI exhibits high in vitro activity against *Stenotrophomonas maltophilia*, an opportunistic MDR pathogen that has emerged as a major cause of HABP and bloodstream infections [300,301,302]. The addition of AVI restored ATM susceptibility in 98% of ATM-nonsusceptible clinical isolates of *S. maltophilia* compared to 61%, 71%, and 15% with CLAV, REL, and VAB, respectively [301].

The FDA approved ATM-AVI (in combination with metronidazole) in February 2025 for treatment of adults with cIAI who have limited or no alternative treatment options [303]. Prior to FDA authorization, the EMA approved ATM-AVI for the treatment of cIAI, HABP, and cUTI caused by MDR bacterial agents [304]. ATM-AVI fills a critical gap in newly developed first-line therapies targeting the increasing problem of challenging CRE infections in the hospital setting, as it is the only approved BL/BLI combination that effectively inhibits MBL-producing Enterobacterales.

Currently, mutational resistance to ATM-AVI among Enterobacterales and non-fermenting organisms appears to be relatively low compared to CAZ-AVI [305], although decreased in vitro susceptibility and/or resistance to ATM-AVI has been reported predominantly among MBL-producing *E. coli* and very infrequently among *K. pneumoniae*, *E. cloacae*, and *S. maltophilia* [197,231,301,306,307,308,309,310]. ATM-AVI resistance among clinical isolates of NDM or non-MBL *E. coli* is associated with genetic alterations to PBP3, the target of ATM, involving the insertion of 4-amino-acid mutations (YRIN, YRIK, YRIP or TIPY) [231,307,308,310,311,312,313,314]. Potential mechanisms of resistance to ATM-AVI in clinical isolates of other Enterobacterales include decreased drug permeability due to outer-membrane porin mutations, upregulation of efflux pump expression, and high-level overexpression of β-lactamases like AmpC [304,306,307]. The selection of clinical isolates resistant to ATM-AVI will likely continue as the clinical use of this novel broad-spectrum combination becomes more widespread, potentially compromising its efficacy. The continuous development of new investigational combination drugs will be critical for maintaining pipeline robustness and armamentarium sustainability as resistance emerges to antibiotics in current clinical use.

### 4.7. Investigational BL/BLI Combinations in Clinical Development

Various innovative BLI combinations in the drug development pipeline have recently completed Phase 3 or Phase 1 clinical trials (Table 2). Additional context is provided below for late-phase clinical trials evaluating cefepime in combination with TAN, ZID, and NAC.

Compared to other investigational agents listed in Table 2, CFP-TAN is most advanced in terms of clinical development, supported by robust published Phase 3 data. The CERTAIN-1 study was a randomized, double-blind, active-controlled Phase 3 study that assessed safety and efficacy of CFP-TAN compared to MER in pathogen eradication and symptomatic response in adult patients with cUTIs, including AP [235,315]. The primary composite endpoint was both microbiologic success (defined as eradication of all baseline Gram-negative uropathogens) and clinical success (defined as symptomatic resolution) in the microbiologic intention-to-treat (microITT) population (436 patients) out of a total of 661 randomized patients [235]. Composite success at test-of-cure (TOC) was achieved in 70.6% of patients in the CFP-TAN group and 58.0% in the MER group, indicating that CFP-TAN was superior to MER. When primary outcomes were assessed by baseline pathogen resistance, composite success occurred in 7/8 (87.5%) patients with CRE and 8/9 (88.9%) patients with Enterobacterales producing a carbapenemase (5 OXA-48 family, 2 KPC-3, 2 NDM-1) [315]. Among the 437 baseline Enterobacterales pathogens identified in the CERTAIN-1 trial, 38.2% were MDR, while only 2.3% were carbapenem-resistant [315]. This Phase 3 trial demonstrated promise for the use of CFP-TAN in eradicating MDR Enterobacterales in vivo, but the limited representation of CRE among enrolled pathogens restricts the clinical generalizability of the observed superiority of CFP-TAN versus MER to the treatment of CRE infections in clinical practice. Additionally, CFP-TAN superiority was demonstrated in a primarily cUTI/AP patient population with a carbapenem comparator rather than with the best available therapy for resistant infections.

ZID and NAC exhibit intrinsic antibacterial activity due to their ability to bind PBP2 with high affinity. Both agents exert an “enhancer effect” when combined with a β-lactam antibiotic targeting PBP3, thereby improving activity potency against carbapenem-producing organisms [239,316]. The CFP-ZID, CFP-NAC, and ATM-NAC combinations, while mechanistically promising based on in vitro data, are clinically less mature compared to CFP-TAN. The Phase 3 CFP-ZID trial was a randomized, double-blind, multicenter, non-inferiority study of 528 hospitalized patients with cUTI or AP [317]. Primary outcome measures were clinical cure and microbiologic eradication at TOC. The active comparator was MER. In the INTEGRAL-1 Phase 3 trial, the safety and efficacy of CFP-NAC or ATM-NAC were compared to IMI/cilastatin in the treatment of cUTI or AP in 614 enrolled patients [318]. This multicenter, randomized, double-blind study measured the proportion of patients who achieved composite clinical and microbiologic success at TOC. In the INTEGRAL-2 Phase 3 trial, a multicenter, randomized, single-blind, parallel group study was conducted to evaluate the efficacy of CFP-NAC or ATM-NAC, compared with the best available therapy, in the treatment of patients with cUTI, AP, HABP, VABP, or cIAI due to CRE [319]. The primary efficacy endpoint was overall treatment success at TOC across all infection types. The small sample size (126 patients) in the CRE-focused trial arm, however, limits the statistical power for demonstrating a clinical benefit of CFP-NAC or ATM-NAC in treating infections caused by CRE infections and carbapenemase-producing Enterobacterales. The clinical data for carbapenem-resistant Gram-negative pathogens are limited across all these Phase 3 programs, and the clinical effectiveness of CFP-TAN, CFP-ZID, CFP-NAC, and ATM-NAC against real-world CRE prevalence still needs to be rigorously established.

## 5. Comparative Inhibitor Spectra: Key Gaps in Coverage

**Figure 4:**
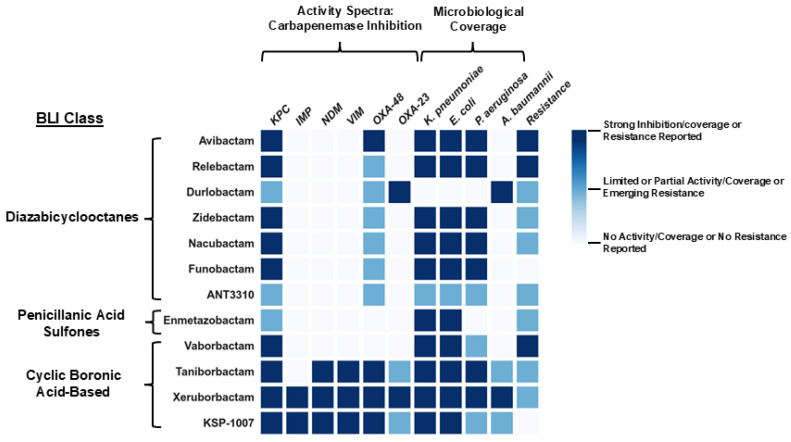
Heatmap of Inhibitory Spectrum and Microbiological Coverage Gaps Among Approved and Investigational BLIs. (Figure created in https://BioRender.com).

Clinical therapeutic decisions are guided by key antimicrobial spectrum characteristics. Figure 4 graphically presents a comparative analysis of critical gaps in the inhibitory spectrum and microbiological coverage of approved and experimental next-generation BLIs. Of the older next-generation and FDA-approved β-lactamase inhibitors, AVI, REL, and VAB potently inhibit serine-based class A KPCs; however, only AVI demonstrates clinically useful inhibitory activity against OXA-48-producing Enterobacterales but not against OXA-producing carbapenem-resistant *A. baumannii*, a critical priority pathogen [198,320]. Except for DUR and several investigational ultrabroad inhibitors (TAN, XER, and KSP-1007), the novel BLIs lack inhibitory activity against *Acinetobacter* OXA carbapenemases such as OXA-23, OXA-24/40, and OXA-58. In addition, approved inhibitors and Phase 3 DBOs lack demonstrated activity against MBLs (Figure 4). Infections caused by MBL-producing organisms are therapeutically problematic because treatment options are severely restricted. While AVI in combination with ATM inhibits MBL-producing Enterobacterales, this inhibition is indirect and due to the inherent stability of ATM against MBL hydrolysis [297,298]. Therefore, two critical gaps in the activity spectrum of approved novel BLIs are the lack of broad, direct inhibition of MBLs and inadequate coverage of OXA-producing *A. baumannii*. Several BL/BLI combinations in Phase 1–3 clinical development aim to address these exigencies in the anti-carbapenemase armamentarium. For example, a key expansion of the XER activity spectrum is its potent in vitro inhibition of major B1-type MBLs (NDM, VIM, and IMP) [96]; however, clinical efficacy has yet to be established. Although XER fills an important gap in the TAN activity spectrum by providing activity against IMP, the recently reported emergence of XER-resistant IMP variants [e.g., IMP-6 (S262G), IMP-10 (V67F), IMP-14 (multiple amino acid substitutions), and IMP-26 (V67F)] is concerning [262]. In contrast, TAN inhibits NDM and VIM but lacks activity against IMP [261]. Resistance driven by newly evolved carbapenemase variants under selective pressure, as well as organism-specific differences (involving intrinsic multiple non-enzymatic resistance mechanisms), will likely continue to impact the durability of the activity spectra of BLIs presently in clinical development.

## 6. Conclusions

Infections caused by carbapenemase-producing Enterobacterales and non-fermentative GNB (e.g., *P. aeruginosa* and *A. baumannii*) are associated with significant mortality due to failed treatment regimens, particularly among immunocompromised patients in healthcare settings. Genetic diversity is a key determinant of the successful global dissemination of carbapenemases, which include multiple β-lactamase classes and enzyme variants with distinct catalytic efficiencies. New carbapenemase variants continue to evolve under antibiotic selective pressure and horizontal gene transfer, acquiring mutations that enhance hydrolytic activity and/or confer resistance to BLIs. This extensive genetic plasticity complicates both epidemiological surveillance and treatment of carbapenem-resistant infections. While the development of next-generation BL/BLI combinations has improved the therapeutic management of infections caused by carbapenemase-positive GNBs, inhibitor coverage across the full diversity of enzyme classes and variants remains incomplete. Addressing this complex issue will require a multifaceted strategy that integrates continuous global and regional surveillance, robust antimicrobial stewardship, prioritization of prescribed novel BL/BLI therapy only in warranted cases, and expansion of the BLI development pipeline. Microbial resistance is an inevitable and ongoing challenge that will always drive innovation and development in drug discovery. Fully implementing artificial intelligence tools, particularly machine learning and deep learning algorithms, into antimicrobial drug pipelines will help keep pace with continuously evolving microbial resistance and address critical coverage gaps in the treatment of carbapenem-resistant infections.

## Figures and Tables

**Table 1:** Comparison of Clinically Relevant Carbapenemase Types in Gram-Negative Bacteria.

Ambler Class (Catalytic Center)	Carbapenemase Family	Substrate Profile	Inhibitors	Predominant Host Species	Variants	References
A * (Serine)	SME	PCNs, CEPs (not extended-spectrum), CARBs, ATM (weak activity)	CLAV, TAZO, AVI; variable inhibition by newer BLIs (limited data)	*Serratia marcescens* (almost exclusively)	SME-1–5; SME- 6 (*Serratia ureilytica*)	[41,42,43,44,45]
	IMI/Nmc-A	PCNs, CEPs (not extended-spectrum), CARBs	CLAV, AVI	*Enterobacter cloacae* complex	IMI-1–IMI-24, Nmc-A	[41,42,43]
	KPC	PCNs, CEPs, CARBs, ATM	AVI, VAB, REL; variable inhibition in vitro by classical BLIs (e.g., CLAV, TAZO, SUL), insufficient to restore clinical susceptibility	*Klebsiella pneumoniae* (primary host), *Escherichia coli*, & other Enterobacterales	290 total; KPC-2 & KPC-3 most prevalent globally	[46,47,48,49]
B1 ** (Zn1, Zn2)	IMP	Broad β-lactam hydrolysis including CARBs; no activity against ATM	EDTA & other metal chelators	Enterobacterales, *Pseudomonas aeruginosa*	107 total; IMP-1 (Japan) & IMP-4 (Australia) common	[50,51,52,53]
	NDM	Broad β-lactam hydrolysis including CARBs; no activity against ATM	EDTA & other metal chelators	*K. pneumoniae*, *E. coli*; *Acinetobacter* & *Pseudomonas* spp.	96 total; NDM-1, NDM-4, NDM-5, NDM-7 most common	[36,42,54,55]
	VIM	Broad β-lactam hydrolysis including CARBs; no activity against ATM	EDTA & other metal chelators	*P. aeruginosa*, Enterobacterales (particularly *K. pneumoniae*, *E. coli*, *Enterobacter* spp.)	94 total; VIM-1 & VIM-2 most frequently identified	[36,56,57,58]
D ^#^ (Serine)	OXA-23	OX, PCNs, CARBs (weak activity); kinetic variation across families	Poor inhibition by CLAV, SUL, TAZO & EDTA; AVI (variable activity) & often limited against many OXA types	*Acinetobacter baumannii*	55 total; OXA-23 dominant	[59,60,61,62,63]
	OXA-24/40	OX, PCNs, CARBs (weak activity)	Poor inhibition by CLAV, SUL, TAZO & EDTA; AVI (variable activity)	*A. baumannii*	15 total; OXA-24/40 & OXA-72 prevalent	[59,64]
	OXA-51	OX, PCNs, CARBs (weak activity)	Poor inhibition by CLAV, SUL, TAZO & EDTA; AVI (variable activity)	*A. baumannii*	398 total; OXA-66, OXA-65, & OXA-69 dominant	[59,65,66]
	OXA-58	OX, PCNs, CARBs (weak activity)	Poor inhibition by CLAV, SUL, TAZO & EDTA; AVI (variable activity)	*A. baumannii*	8 total; OXA-58 most widely reported; OXA-96	[59,67]
	OXA-48	OX, PCNs, CARBs (weak activity)	AVI	*K. pneumoniae*, *E. coli*, *E. cloacae* complex	23 total; OXA-48, OXA-181, OXA-232 & OXA-244 clinically relevant	[59,68,69,70]

* Ambler Class A carbapenemases are serine β-lactamases that hydrolyze carbapenems using a serine-based acylation–deacylation mechanism. ** Ambler Class B1 carbapenemases, a subclass of the metallo-β-lactamases, use one or two Zn^2+^ ions to activate a water molecule for direct nucleophilic attack on the β-lactam ring, enabling potent carbapenem hydrolysis. ^#^ Ambler Class D carbapenemases are oxacillinases that also employ serine-based acylation but rely on a carbamylated lysine to mediate deacylation. This mechanism leads to slower overall, but still clinically significant, rates of carbapenem hydrolysis. It is important to note that not all OXA variants hydrolyze carbapenems; only a subset of OXA family variants are characterized as carbapenem-hydrolyzing Class D β-lactamases. SME, *Serratia marcescens* enzyme; IMI/NmcA, Imipenem-hydrolyzing β-lactamase/non-metallo-carbapenemase A; KPC, *Klebsiella pneumoniae* carbapenemase; IMP, Imipenemase; NDM, New Delhi metallo-β-lactamase; VIM, Verona integron-encoded metallo-β-lactamase; OXA, Oxacillinase; PCNs, Penicillins; CEPs, Cephalosporins; CARBs, Carbapenems; ATM, Aztreonam; CLAV, Clavulanate; TAZO, Tazobactam; SUL, Sulbactam; AVI, Avibactam; BLIs, Beta (β)-lactamase inhibitors; REL, Relebactam; VAB, Vaborbactam; OX, oxacillin; EDTA, Ethylenediaminetetraacetic acid.

**Table 2:** Recently Approved and Emerging BL/BLI Combinations in Clinical Development.

Drug Name	Novel BLI Structure	US FDA Status or Clinical Phase * (as of February 2026)	Mechanism of Action	Approved or Investigated Indications	Key Enzyme & Bacterial Targets	Limitations in Coverage Spectrum	Key Sources
Ceftazidime-Avibactam (Avycaz^®^)	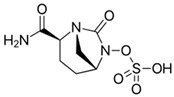	Approved February 2015; expanded approval February 2018	3rd-generation cephalosporin (CAZ) & non-β-lactam/DBO BLI (AVI)	cUTI & cIAI (in combination w/MET) [initial approval]; HABP & VABP (expanded approval)	KPC, ESBL, AmpC, OXA-48 Enterobacterales, CR *Pseudomonas aeruginosa* strains	No activity against MBLs (NDM, VIM, IMP); low barrier to resistance, often treatment-selected	[198,199,200,201,202,203]
Meropenem-Vaborbactam (Vabomere^®^)	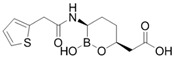	Approved August 2017	Carbapenem (MER) & non-β-lactam/cyclic boronic acid BLI (VAB)	cUTIs including AP in adults	KPC, other class A serine BLs Enterobacterales	No activity against class D (OXA) and MBL producers; limited non-fermenter coverage (*Pseudomonas* & *Acinetobacter* spp.)	[198,204,205,206,207,208]
Imipenem/Cilastatin/Relebactam (Recarbrio^®^)	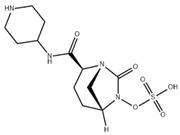	Approved July 2019 & June 2020	Carbapenem (IMI), renal DHP-I inhibitor (Cilastin) & DBO BLI (REL)	cUTIs, cIAIs, HABP, and VABP	Class A serine BLs (ESBL, KPC) and Class C (AmpC) KPC-positive Enterobacterales, some CR *P. aeruginosa*	No activity against MBL (NDM, VIM, IMP) producers; limited/no activity against OXA-48-like CRE; emerging resistance due to porin loss, KPC allele mutations, and/or increased *bla*_KPC_ copy number	[198,209,210,211,212,213,214]
Sulbactam-Durlobactam (Xacduro^®^)	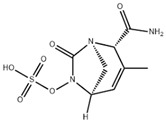	Approved May 2023	Non-β-lactam/β-lactamase inhibitor (SUL) & non-β-lactam/DBO BLI (DUR)	HABP & VABP caused by susceptible isolates of *Acinetobacter baumannii*-*calcoaceticus* complex in adults	*Acinetobacter baumannii* and other ABC species, including CR, MDR & XDR; limited activity against CR-GNBs	Durlobactam does not inhibit class B MBLs; not broadly active against Enterobacterales or *Pseudomonas aeruginosa* carbapenemases	[215,216,217,218,219]
Cefepime-Enmetazobactam (Exblifep^®^)	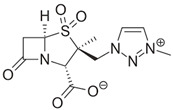	Approved February 2024 (US FDA) & March 2024 (EMA)	4th-generation cephalosporin (CFP) & non-β-lactam/penicillanic acid sulfone BLI (ENM); both agents are zwitterionic	cUTIs, including AP (FDA approval) cUTIs (+ AP), HABP/ VABP, & BSI (EMA approval)	CTX-M, TEM, & SHV ESBLs; other class A β-lactamases; some KPCs (not reliably susceptible) & OXA-48 (in vitro data only) ESBL-producing Enterobacterales	ENM has no inhibitory activity against class B MBLs; no additional coverage for *P. aeruginosa* and *A. baumannii* over CEF; spectrum gaps also include MRSA, enterococci & anaerobes	[220,221,222,223,224,225]
Aztreonam-Avibactam (Emblaveo^™^)	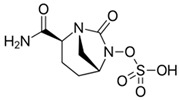	Approved February 2025	Monobactam (ATM) & non-β-lactam/DBO BLI (AVI)	Combined w/MET for cIAIs in adults w/limited or no alternative treatment options. cIAI, HABP, VABP & cUTI (EMA only, April 2024)	Class A (ESBL, KPC), Class B MBLs (inhibitory activity due to ATM partner), Class C (AmpC), & Class D (OXA-48-like) CRE (high in vitro activity); *S. maltophilia*; *P. aeruginosa* (less potent activity)	Limited *Acinetobacter*, Gram-positive & anaerobe coverage; emerging resistance, especially among *E. coli* isolates, due to PBP3 modifications	[197,201,226,227,228,229,230,231]
Cefepime-Taniborbactam (VNRX-5133)	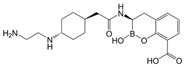	Phase 3 (CERTAIN-1) completed 14 December 2021	4th-generation cephalosporin (CFP) & bicyclic boronic acid BLI (TAN)	cUTI + AP trial (adults) vs. comparator drug MER (NCT03840148 **)	Class A (ESBL, KPC), Class B (VIM, NDM), Class C (AmpC), Class D (OXA-48-like) Enterobacterales (CR, MDR) & *P. aeruginosa* (CR, MDR)	Not currently approved; IMP MBLs not inhibited by TAN; limited activity against *Acinetobacter*, Gram-positives, anaerobes; emergence of resistant variants NDM-9, NDM-30, & VIM-83	[232,233,234,235,236,237,238]
Cefepime-Zidebactam (WCK 5222)	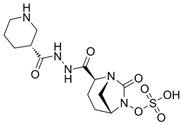	Phase 3 completed 25 November 2024	4th-generation cephalosporin (CFP) & DBO BLI with PBP2 binding (ZID)	cUTI + AP trial (adults) vs. comparator MER (NCT04979806)	ESBL, KPC, MBLs (IMP, VIM, NDM), AmpC, OXA-48 MDR Enterobacterales, *P. aeruginosa* including CR	Clinical results pending; limited activity against *Acinetobacter* spp.; reported treatment-emergent resistance in *P. aeruginosa*	[239,240,241,242,243,244]
Aztreonam-Nacubactam (OP0595/RG6080)	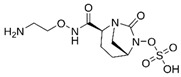	Phase 3 completed 26 November 2024 Phase 3 completed 1 September 2025	Monobactam (ATM) & DBO BLI with PBP2 binding activity and enhancer effect (NAC)	cUTI + AP trial (adults) vs. Cefepime-nacubactam & MER (NCT05887908) CRE infections trial (adults) vs. Cefepime-nacubactam & BAT (NCT05905055)	MBLs (NDM, IMP), serine β-lactamases CRE, *S. maltophilia*	No regulatory approvals as of February 2026	[245,246,247,248,249]
Cefepime-Nacubactam (OP0595/RG6080)	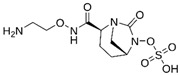	Phase 3 completed 26 November 2024 Phase 3 completed 1 September 2025	4th-generation cephalosporin (CFP) & DBO BLI with PBP2 binding activity and enhancer effect (NAC)	cUTI + AP trial (adults) vs. Aztreonam-nacubactam & MER (NCT05887908) CRE infections trial (adults) vs. Aztreonam-nacubactam & BAT (NCT05905055)	KPC, OXA, MBLs, ESBL, AmpC CRE, Enterobacterales, *P. aeruginosa*, *S. maltophilia*	No regulatory approvals as of February 2026	[245,247,248,249]
Imipenem-Cilastatin-Funobactam (XNW4107)	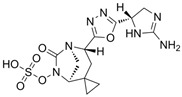	Phase 3, status unknown, estimated completion December 2025	Carbapenem (IMI), renal DHP-I inhibitor (Cilastin) & DBO BLI that potentiates IMI (funobactam)	cUTI + AP trial (adults) vs. MER (NCT05204368)	KPC, some MBLs, OXA, ESBL CRE, MDR Enterobacterales, MDR *P. aeruginosa*	Clinical results pending; incomplete MBL coverage; limited activity against *Acinetobacter*	[250,251,252,253]
Meropenem-Nacubactam (OP0595/RG6080)	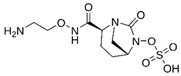	Phase 1 completed 10 August 2017	Carbapenem (MER) & DBO BLI with PBP2 binding activity and enhancer effect (NAC)	Non-randomized, open-label trial; intrapulmonary lung penetration of NAC in healthy adults (NCT03182504); GNB infections	ESBL, KPC, class B NDM, AmpC, OXA-48 MDR Enterobacterales	Early clinical development; safety/efficacy not established	[245,248,254,255]
Meropenem-KSP-1007	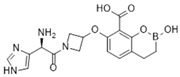	Phase 1 completed 1 October 2022	Carbapenem (MER) & bicyclic boronic acid BLI (KSP-1007)	First-in-human, randomized, double-blind clinical trial (NCT05226923) to treat CR-GNB infections	Broad inhibition of Ambler class A, B, C & D enzymes: KPC, MBLs (NDM, VIM, IMP, except IMP-6), AmpC, & OXAs (including OXA-48, *Acinetobacter* OXAs) CRE, *A. baumannii* (plus OXA producers) & *P. aeruginosa* (2-fold MER MIC reductions observed)	Early clinical development; safety/efficacy not established	[256]
Ertapenem-Zidebactam (WCK 5222)	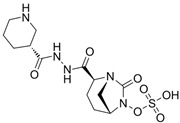	Phase 1 completed 3 November 2023	Carbapenem (ETP) & DBO BLI with PBP2 binding (ZID)	Single-center trial (NCT05645757); bacterial infections	*E. coli* w/AmpC, ESBLs, KPC, MBLs, or OXA-48 (>90% inhibited)	Early clinical development; safety & PK studied but efficacy not established	[257]
Xeruborbactam (QPX7728) Combinations	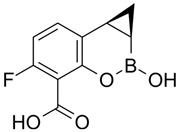	Phase 1 trials in combination w/Ceftibuten (NCT06079775 completed 5 January 2025) & Cefiderocol (NCT06547554 completed 27 October 2025)	Oral dosage of 3rd-generation cephalosporin (ceftibuten) & bicyclic boronic acid BLI (XER) Siderophore cephalosporin (CFD) & bicyclic boronic acid BLI (XER)	Both Phase 1 trials studied the safety, tolerability, & PK	Potential Pan-BLI (ultrabroad spectrum); inhibits KPC, MBLs (NDM, VIM, IMP), class D OXA enzymes (e.g., OXA-48 in Enterobacterales and OXA-23/OXA-40 in *A. baumannii*) as well as other class A and class C β-lactamases	Early clinical development; safety & PK studied but efficacy not established; reported XER-resistant IMP variants (IMP-6, IMP-10, IMP-14, IMP-26)	[258,259,260,261,262]
Meropenem-ANT3310	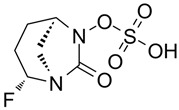	Phase 1 completed 19 August 2025	Carbapenem (MER) & DBO serine-BLI (ANT3310)	Open-label, single-center trial to determine MER-ANT3310 penetration into the lung in healthy adults (NCT06916156)	KPC & OXA (including OXA-23, OXA-24/40, OXA-51, OXA-58); potentiates activity of MER against CR *A. baumannii* and Enterobacterales	Early clinical development; safety/efficacy not established	[263,264,265]

* Clinical phase status obtained from ClinicalTrials.gov. ** ClinicalTrials.gov ID. FDA, Food and Drug Administration; BLI, β-lactamase inhibitor; DBO, diazabicyclooctane; PBP2/3, Penicillin-binding protein 2/3; BAT, Best available therapy; CAZ, Ceftazidime; AVI, Avibactam; MER, Meropenem; VAB, Vaborbactam; IMI, Imipenem; DHP-I, Dehydropeptidase-I inhibitor; REL, Relebactam; SUL, Sulbactam; DUR, Durlobactam; CFP, Cefepime; ENM, Enmetazobactam; ATM, Aztreonam; ETP, Ertapenem; MET, Metronidazole; TAN, Taniborbactam; ZID, Zidebactam; NAC, Nacubactam; XER, Xeruborbactam; MBLs, Metallo-β-lactamases; CR, Carbapenem-resistant; GNB, Gram-negative bacteria; MDR, Multidrug-resistant; XDR, Extensively drug-resistant; CRE, Carbapenem-resistant Enterobacterales; cUTIs, Complicated urinary tract infections; AP, Acute pyelonephritis; cIAIs, Complicated intra-abdominal infections; HABP, Hospital-acquired bacterial pneumonia; VABP, Ventilator-associated bacterial pneumonia; BSI, Bloodstream infection; ESBL, Extended-spectrum β-lactamase; KPC, *Klebsiella pneumoniae* carbapenemase; IMP, Imipenemase; NDM, New Delhi metallo-β-lactamase; VIM, Verona integron-encoded metallo-β-lactamase; OXA, Oxacillinase; EMA, European Medicines Agency; MRSA, Methicillin-resistant *Staphylococcus aureus*.

## Data Availability

No new data were created or analyzed in this study.

## Abbreviations

The following abbreviations are used in this manuscript:

ABC	*Acinetobacter baumannii-calcoaceticus* complex
AI	Artificial intelligence
AP	Acute pyelonephritis
ATM	Aztreonam
ATM-AVI	Aztreonam-Avibactam
AVI	Avibactam
BAT	Best available therapy
BL/BLI	β-lactam/β-lactamase inhibitor
BLI	β-lactamase inhibitor
BPPL	Bacterial Priority Pathogens List
BSI	Bloodstream infection
C	Chromosomal
CARB	Carbapenem
CAZ	Ceftazidime
CAZ-AVI	Ceftazidime-Avibactam
CDC	Centers for Disease Control and Prevention
CEP	Cephalosporins
CFP	Cefepime
CFP-ENM	Cefepime-Enmetazobactam
CI	Confidence interval
cIAI	Complicated intra-abdominal infection
CLAV	Clavulanate
CP	Carbapenemase-producing
CRAB	Carbapenem-resistant *Acinetobacter baumannii*
CRE	Carbapenem-resistant Enterobacterales
CR-GNB	Carbapenem-resistant Gram-negative bacteria
CRKP	Carbapenem-resistant *Klebsiella pneumoniae*
CRPA	Carbapenem-resistant *Pseudomonas aeruginosa*
cUTI	Complicated urinary tract infection
DBO	Diazabicyclooctane
DHP-I	Dehydropeptidase-I inhibitor
DUR	Durlobactam
EDTA	Ethylenediaminetetraacetic acid
EMA	European Medicines Agency
ENM	Enmetazobactam
ESBL	Extended-spectrum β-lactamase
ETP	Ertapenem
FDA	Food and Drug Administration
HABP	Hospital-acquired bacterial pneumonia
ICU	Intensive care unit
IMI	Imipenem
IMI/Nmc-A	Imipenemase/non-metallo-carbapenemase A
IMI-REL	Imipenem-Cilastatin-Relebactam
IMP	Imipenemase metallo-β-lactamase
IS	Insertion sequence
KPC	*Klebsiella pneumoniae* carbapenemase
MBL	Metallo-β-lactamase
MDR	Multidrug-resistant
MER	Meropenem
MER-VAB	Meropenem-Vaborbactam
MET	Metronidazole
MIC	Minimum inhibitory concentration
MRSA	Methicillin-resistant *Staphylococcus aureus*
NAC	Nacubactam
NCBI	National Center for Biotechnology Information
NDM	New Delhi metallo-β-lactamase
OR	Odds ratio
OX	Oxacillin
OXA	Oxacillinase
P	Plasmid
PBP2/3	Penicillin-binding protein 2/3
PCN	Penicillin
REL	Relebactam
RR	Relative risk
SME	*Serratia marcescens* enzyme
SUL	Sulbactam
SUL-DUR	Sulbactam-Durlobactam
TAN	Taniborbactam
TAZO	Tazobactam
TOC	Test of cure
VAB	Vaborbactam
VABP	Ventilator-associated bacterial pneumonia
VIM	Verona integron-encoded metallo-β-lactamase
XDR	Extensively drug-resistant
XER	Xeruborbactam
ZID	Zidebactam
WHO	World Health Organization
